# Adipose-Derived Mesenchymal Stem Cells Ameliorating *Pseudomonas aeruginosa*–induced Acute Lung Infection *via* Inhibition of NLRC4 Inflammasome

**DOI:** 10.3389/fcimb.2020.581535

**Published:** 2021-01-08

**Authors:** Lu-lu Li, Ying-gang Zhu, Xin-ming Jia, Dong Liu, Jie-ming Qu

**Affiliations:** ^1^ Department of Respiratory and Critical Care Medicine, Ruijin Hospital, School of Medicine, Shanghai Jiao Tong University, Shanghai, China; ^2^ Institute of Respiratory Diseases, School of Medicine, Shanghai Jiao Tong University, Shanghai, China; ^3^ Department of Respiratory and Critical Care Medicine, Huadong Hospital, Fudan University, Shanghai, China; ^4^ Clinical Translational Research Center, Shanghai Pulmonary Hospital, Tongji University School of Medicine, Shanghai, China

**Keywords:** inflammasome, NLRC4, mesenchymal stem cells, *Pseudomonas aeruginosa*, acute pulmonary infection

## Abstract

**Background:**

*Pseudomonas aeruginosa* (PA) is one of the most common Gram-negative bacteria causing hospital-acquired pulmonary infection, with high drug resistance and mortality. Therefore, it is urgent to introduce new non-antibiotic treatment strategies. Mesenchymal stem cells (MSCs), as important members of the stem cell family, were demonstrated to alleviate pathological damage in acute lung injury. However, the potential mechanism how MSC alleviate acute lung infection caused by PA remains unclear.

**Objective:**

The purpose of this study was to investigate the effects of Adipose-derived mesenchymal stem cells (ASCs) on acute pulmonary infections and the possible mechanisms how ASCs reduce pulmonary inflammation induced by PA.

**Methods:**

The therapeutic and mechanistic effects of ASCs on PA pulmonary infection were evaluated respectively in a murine model as well as in an *in vitro* model stimulated by PA and co-cultured with ASCs.

**Results:**

1. ASCs treatment significantly reduced the bacterial load, inflammation of lung tissue and histopathological damage by PA. 2. PA infection mainly activated Nod-like receptor containing a caspase activating and recruitment domain 4 (NLRC4) inflammasome in the lung of mice. ASCs attenuated acute lung infection in mice by inhibiting NLRC4 inflammasome activation. 3. NLRC4^−/−^ mice showed a significant improvement in survival rate and lung bacterial load after PA infection. 4. ASCs mainly increased expression and secretion of STC‐1 in response to PA‐stimulated NLRC4 inflammasome activation.

**Conclusions:**

PA infection attenuated macrophage phagocytosis through activation of NLRC4 inflammasome in macrophages, which eventually led to pulmonary inflammatory damage in mouse; ASCs reduced the activation of NLRC4 inflammasome in macrophages induced by PA infection, thereby increasing the phagocytic ability of macrophages, and ultimately improving lung tissue damage in mouse; ASCs may inhibit NLRC4 inflammasome through the secretion of STC-1.

## Introduction

*Pseudomonas aeruginosa* (PA) is one of the Gram-negative bacteria which can cause acute lung infection in immunocompromised humans and can be always isolated from patients suffering from hospital-acquired pneumonia. PA infection is highly prone to drug resistance, and the means of treatment are scarce, the mortality rate is extremely high despite improvements in supportive care and antibiotic use (Neuhauser et al., 2003; Shorr, 2009; Von Baum et al., 2010; Song et al., 2012; Peng et al., 2014; Wang et al., 2014). If PA-infected patients have acute respiratory distress syndrome (ARDS), the mortality rate can reach up to 70% (Song et al., 2014). To date, several studies have demonstrated that the administration of mesenchymal stem or stromal cell (MSC) attenuated lung inflammation and histological damage while improving recovery from acute lung injury (ALI) caused by endotoxin (Gupta et al., 2007; Mei et al., 2007; Xu et al., 2007; Xu et al., 2008; Krasnodembskaya et al., 2010), *E. coli* (Gupta et al., 2012; Lee et al., 2013) or sepsis (Nemeth et al., 2009; Gonzalez-Rey et al., 2009). Previous studies in our group (Mao et al., 2015) have also reported that MSC can effectively reduce inflammatory damage and improve the survival rate in lung in a mouse model of pulmonary infection caused by PA. Our group (Zhu et al., 2013) have summarized that the potential therapeutic mechanisms of MSC in models of ALI appear to be based on immunomodulation of inflammatory cells such as alveolar macrophages, improvement in alveolar fluid clearance, secretion of anti-microbial peptides/proteins and suppression of lung protein permeability. However, an understanding of the underlying mechanisms surrounding therapeutic benefit of MSC remains incomplete.

Recent findings have demonstrated that MSC actively interact with components of the innate immune system and that, through these interactions, they display anti-inflammatory effects (Zhu et al., 2013). As far as we know to date, these MSC immunomodulatory properties are largely due to paracrine effects from secretion of biologic factors. In a context of inflammation, MSC may secrete paracrine factors that influence innate immune cell subpopulations such as macrophages to suppress the inflammatory response (Chen et al., 2008; Fontaine et al., 2016). Although the immunomodulatory effects of MSC paracrine factors have been clarified *in vitro* assays and *in vivo* murine models, the mechanisms regulating target cells such as macrophages through expression of these factors remain only partially understood.

Recent studies have revealed that inflammasomes may play a crucial role in the pathogenesis of acute inflammatory damage. Inflammasomes are cytosolic multiprotein complexes that are present mainly in myeloid cells including macrophages and dendritic cells (Chen et al., 2014). They proteolytically activate caspase‐1 in response to cellular “danger” signals. Activated caspase‐1, in turn, processes proinflammatory cytokines including pro‐interleukin IL-1β and pro‐interleukin IL-18 into the biologically active and secreted forms. IL‐1β and IL‐18 act on neighboring non-immune and immune cells to initiate and orchestrate inflammatory responses (Heng et al., 1995; Poyet et al., 2001; Ravi Kumar et al., 2018). To date, four inflammasomes have been identified based on their structure and recognition signals: including NLRP3, NLRC4, AIM2 and NLRP1. Among them, Nod-like receptor protein 3 (NLRP3) and Nod-like receptor containing a caspase activating and recruitment domain 4 (NLRC4) inflammasomes are mostly studied. NLRP3 inflammasome was mainly activated by excess PAMP such as lipopolysaccharide (LPS) and peptidoglycan. MSC have been shown to inhibit NLRP3 inflammasome activation in macrophages to relieve excessive inflammation (Lee et al., 2009; Oh et al., 2014; He et al., 2016; Sun et al., 2017; Park et al., 2018). NLRC4 inflammasome in macrophages can be activated and subsequently induce downstream inflammatory response upon sensing presence of PA infection (Sutterwala et al., 2007; Miao et al., 2016). However, whether MSC can alleviate PA-induced acute lung infection by affecting the activation of NLRC4 inflammasome remains unclear, and the mechanisms underlying the effects of MSC on NLRC4 inflammasome activated by PA infection remain partially elucidated.

Compared to bone marrow-derived mesenchymal stem cells, adipose-derived mesenchymal stem cells (ASCs) is relatively easy to obtain, a large amount of ASCs can be extracted from adipose tissue obtained through surgical minimally invasive surgery, and considered as an ideal and suitable origin of mesenchymal stem cells in experimental research. In this study, we hypothesized that Adipose-derived mesenchymal stem cells (ASCs) may attenuate PA-induced acute lung infection by inhibiting activation of the NLRC4 inflammasome in macrophages, and the substantial secretion of paracrine soluble factors from ASCs could mediate the immunoregulatory effect of ASCs on NLRC4 inflammasome.

## Materials and Methods

### Isolation and Culture of ASCs

Adipose-derived mesenchymal stem cells were isolated from mouse adipose tissue by previously reported methods (Mao et al., 2015). Briefly, inguinal adipose tissues of C57BL/6 mice (7–8 weeks of age) were isolated in Hank’s Balanced Salt Solution (HBSS, 24020117, Life Technologies, USA) and washed three times with HBSS at 280 g to remove blood and liquid. Clear fat’s tissues were cut into 1 mm small fragments and incubated in the solution of HBSS contained 100 units/ml collagenase type I (SCR103, Sigma-Aldrich, USA) in a 37°C water bath stirred at 150 rpm for 60 minutes. After neutralization with fetal bovine serum (FBS, 10091, Gibco Life Technologies, USA), cells were centrifuged at 1500 rpm for 7 minutes, the pellet was washed in HBSS, and filtered through 70 μm then 40 μm nylon mesh. ASCs were cultured in Dulbecco’s Modified Eagle Medium/Nutrient Mixture F-12 (DMEM/F-12, 11330057, Gibco, USA) containing 100 U/ml penicillin/streptomycin and 10% FBS in 75 cm^2^ flasks. The flasks were incubated at 37°C in the humid atmosphere at 5% CO_2_. Non-adherent cells were removed by medium replacement after 24 h. Culture media was changed every 2–3 days. Upon confluence at 80% to 90% density, cells were sub-cultured through 0.05% Trypsin-EDTA (Gibco, 25300054, USA) in 1:2 ratio. Passage 4 of ASCs were used for studies.

### Preparation of *Pseudomonas aeruginosa* Strain

The well-characterized, motile, non-mucoid laboratory strains of PA named as PAO-1 (ATCC-BAA-47; strain HER-1018) from overnight cultures on trypticase soy agar (Difco) plates were grown in 10 ml of trypticase soy broth (Difco) at 37 °C with shaking at 225 rpm and subsequently diluted 1:50 (vol/vol) and grown for 2 to 2.5 h until an optical density (600nm) corresponding to 2 × 10^8^ colony-forming units per 0.5 ml was reached. The bacteria were resuspended in PBS (Hyclone, USA) and washed three times, then were diluted in each specific serum-free medium before infection *in vivo* or added to cells at the concentration indicated *in vitro*.

### PA-induced Acute Pulmonary Infection in Mice

Male specific pathogen-free (SPF) C57BL/6 mice, 7 to 8 weeks of age, were used in all experiments. The mice were purchased from Shanghai ShiLaiKe Experiment Animals Company (Shanghai, China). Acute lung infection was induced by the instillation of PA intratracheally (i.t.). The glottis of mouse was visualized by cold light source, and 40 µl of PA inoculum containing a sublethal dose of 1×10^6^ colony form unit (CFU) bacteria was delivered i.t. using a sterile 24-gauge SURFLOVR Flash I.V. Catheter (TERUMO, Tokyo, Japan). Animals were maintained in a SPF facility and monitored daily by veterinary staff. The Ethical Committee of the Department of Laboratory Animal Science of Shanghai Jiaotong University approved all the experiment protocols.

Animals were anesthetized by intraperitoneal (i.p.) administration of Pentobarbital sodium (250 mg/kg, 0.015 mg/ml, Notlas, China). Different groups of administrations were given simultaneously while acute lung infection was initiated. Groups were designed as follows: (a) ASCs treatment group; (b) L929 treatment group (NCTC clone 929 (L-929) Cell Line was purchased from the Cell Culture Bank of the Chinese Academy of Sciences’ Type Culture Collection Committee/Cell Resource Center, Shanghai Institutes for Biological Sciences, Chinese Academy of Sciences): a fibroblast treatment group as a control; (c) PA (positive control): PBS treatment group; (d) sham (negative control): a group without LPS induction and treatment, while with operations similar to the other groups.

Three hours after instillation of PA, 1 × 10^6^ cells/ml ASCs in 40ul volume were delivered to the lungs with the same method. Twenty-four hours or 36 h post-infection, both broncho-alveolar lavage fluid (BALF) samples or lungs were collected from each mouse for assessment of inflammatory cell counts, cytokine, and protein level measurements and histology as described below.

In separate experiments, WT mice, NLRC4^−/−^ mice, and NLRP3^−/−^ mice were infected by PA respectively. Male specific pathogen-free (SPF) NLRC4^−/−^ mice and NLRP3^−/−^ mice derived from C57BL/6 genetic background, 7 to 8 weeks old, were kindly provided by Professor Jin-fu Xu, Tongji University School of Medicine (NLRP3^−/−^ mice care and use for all experiments was approved by the Harvard Medical Area Standing Committee on Animals of Harvard Medical School. NLRC4^−/−^ mice were provided by Dr. Nunez, University of Michigan) (Xu et al., 2012; Fan et al., 2017).

Mice were intratracheally injected with ASCs or L929 cells 3 h later. The mice were divided into four groups: control group, PA infection group, PA + ASCs treatment group, and PA + L929 control group. Each group was continuously observed to 120 h to record the survival of mice.

### Measurement of Inflammatory Cell Counts, Cytokine, and Bacterial Burden in BALF and Lung Homogenate

Total white blood cell count in BALF was quantified with a hemocytometer. Mouse macrophage inflammatory protein (MIP)-2, tumor necrosis factor (TNF-α), mouse or human stanniocalcin (STC)-1, IL-1β and IL-18 were measured in BALF or lung with ELISA kits (R&D Systems, USA; SAB, China; eBioscience, USA; ABclonal, China). Each specimen (lung, BALF) was plated on cetrimide agar plates using serial 10-folds dilutions. The colonies were counted from culture plates and computed as CFU per whole lung, CFU per milliliter of BALF.

### Histology and Lung Injury Score

Twenty-four hours or 36 h post-infection, both lungs were collected, fixed, sliced and stained with hematoxylin and eosin for microscopic examination. An independent outside examiner experienced in lung histopathological assessment and blinded to group assignment analyzed the lung tissue sections according to the scoring system of the American Thoracic Society (Matute-Bello et al., 2011). The lung injury score (LIS) varies from 0 to 1 where 0 is no injury and 1 is the maximal injury.

### Co-culture of PA-stimulated Macrophages and ASCs

THP-1 cell line, as a human monocytic cell line, was friendly provided by Dr. Zhou (Tongji University School of Medicine) and was differentiated into macrophages as previously described (Daigneault et al., 2010). Briefly, THP-1 cells were maintained at 2×10^6^ cells/ml in RPMI 1640 medium supplemented with 10% FBS and 100 U/ml PS at 37 °C in 5% CO2. Then THP-1 cells were differentiated using phorbol 12-myristate 13-acetate (PMA, 0.3ul/12ml, Sigma-Aldrich) for 48 h. Cells were incubated in fresh RPMI 1640 (10% FCS) after removing the PMA-containing media until further assays. Mouse peritoneal macrophages were acquired from C57BL/6 mice as previously described (Choi et al., 2011). Mouse peritoneal macrophages were cultured in high glucose Dulbecco’s modified Eagle’s medium (DMEM) (Gibco, USA) supplemented with 10% heat-inactivated FBS and 1% PS at 37°C in 5% CO_2_.

For inflammasome activation by PA stimulation, macrophages were seeded at 2×10^6^ cells/ml in twelve-well plates and were treated with PA with MOI(bacteria: cells = 1:1)for 4 h. For co-culture experiments and pretreatment, ASCs were seeded at 1×10^6^ in twelve-well transwell before co-culture with macrophages. Just prior to co-culture, ASCs were washed with PBS three times and co-cultured with macrophages in the macrophage culture medium. The macrophages stimulated by PA were treated with recombinant human STC-1 (rhSTC-1, R&D systems, USA), TSG-6 (rhTSG-6, R&D systems, USA) and IL-RA (rhIL-RA, R&D systems, USA) (10ng/ml, 100ng/ml, 200ng/ml) from PA priming step.

The cell-free supernatants were then collected at each time point after co-incubation to assay for the levels of mouse or human stanniocalcin (STC)-1, IL-1β, and IL-18 with ELISA kits (SAB, China; eBioscience, USA; ABclonal. China).

### Western Blot Analysis

Recovery of protein from cell or tissue supernatants was based on Protein Extraction Solution (RIPA: PMSF = 100:1, Beyotime, China). Clear cell lysates were measured for protein concentration by BCA assay (Beyotime, China). Caspase-1 (p20) was detected with rabbit anti-Caspase-1 monoclonal antibody (CST, USA). NLRC4 was detected with rabbit anti-NLRC4 monoclonal antibody (ECM Biosciences, China). β-actin was detected with rabbit anti-β-actin monoclonal antibody (Abmart, Chin). The membranes were processed with ECL chemiluminescence kit (Thermo Fisher Scientific).

### RNA Isolation and Quantitative Real-Time RT‐PCR

Total RNA in the lung was isolated with Trizol (Invitrogen, Thermo Fisher Scientific, Waltham, MA, USA), and reverse-transcribed by using FastQuant RT Kit (with gDNase) (TIANGEN BIOTECH, China) following the manufacturer’s manual instructions. Single stranded cDNA was then amplified by RT-PCR with specific primers of NLRC4, NLRP3 and GAPDH: mouse NLRC4 gene sequence (5′ to 3′): F CAGGTGGTCTGATTGACAGC, R CCCCAATGTCAGACAAATGA; NLRP3 gene sequence (5′ to 3′): F TGCAGAAGACTGACGTCTCC, R CGTACAGGCAGTAGAACAGTTC; GAPDH gene sequence (5′ to 3′): F CGTCCCGTAGACAAAATGGT, R TTGATGGCAACAATCTCCAC. RT-PCR was performed on an ABI Prism 7000 thermocycler (Applied Biosystems, Thermo Fisher Scientific, Waltham, MA, USA) with SYBR Green PCR Master Mix (Roche, Basel, Switzer- land). For each experiment, samples (n = 5) were run in triplicate. Relative gene expression was calculated using the comparative CT method.

### Cytotoxicity Assay and Phagocytosis of Macrophages

The macrophages were co-cultured with ASCs as described above and were treated with PA with MOI(bacteria: cells = 1:1)for 4 h. Cytotoxicity assay was determined by measuring LDH release in the supernatant according to the manufacturer’s instructions (Promega, USA).

Macrophages were harvested as described above and the ability of macrophages to phagocytose bacteria was measured using green fluorescence protein (GFP)-labeled PA as previously described (Ojielo et al., 2003). Briefly, macrophages were co-cultured with ASCs as described above and were treated with GFP-PA with MOI(bacteria: cells = 1:1)for 1 h. The phagocytosis was quantified with a fluorometer. Fluorescence intensity was proportional to phagocytic ability of macrophages.

### Statistical Analysis

For bacterial burden, comparisons among three or more groups were performed with nonparametric Kruskal-Wallis one-way analysis of variance (ANOVA). Except for bacterial burden, comparisons between two groups were performed using the Student’s t-test, and comparisons among three or more groups were performed with ANOVA and a *post hoc* Bonferroni test. A value of *p* < 0.05 was considered statistically significant. Analyses were performed using GraphPad Prism 7.0 software. Data were shown as mean ± standard deviation.

## Results

### Effect of ASCs on Inflammatory Cell Influx and Cytokine Levels and Histological Severity in PA-induced Acute Pulmonary Infection in Mice

**Figure 1:**
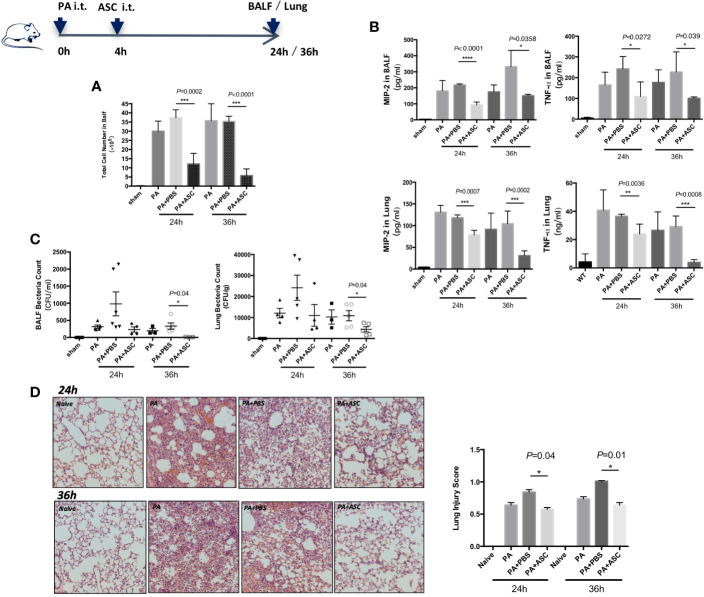
Effect of ASCs on Inflammatory Cell Influx and cytokine levels and Histological Severity in PA-induced acute pulmonary infection in Mice. **(A–D)** The mice were randomly treated and divided respectively into four groups: control group (sham), PA infection group (PA), PA+PBS control group (PA+PBS), and PA+ASC (1×106 cells) treatment group. At 24 or 36 h postinfection, compared with the PA-infected group, the BALF total inflammatory cell count was significantly higher in the PA+PBS group. The expression levels of TNF-α and MIP-2, and the bacterial load in BALF and lung were all significantly increased. Lung tissue damage is more serious, and the pathological score of lung tissue is also significantly increased. When infected mice were treated with ASC, the BALF total inflammatory cell count was significantly reduced. This treatment also reduced the expression levels of TNF-α and MIP-2, and bacterial burdens in lung and BALF. In addition, the pathological damage of lung tissue is significantly alleviated (200×), and the pathological score of lung tissue is also significantly reduced. Data were shown as mean ± SD. ASC, adipose tissue-derived mesenchymal stem cells; BALF, broncho-alveolar lavage fluid; CFU, colony form unit; MIP-2, macrophage inflammatory protein-2; TNF-α, tumor necrosis factor-α.

We have determined that the optimal number of bacteria in PA infection was 1 × 10^6^ CFU (**Supplementary Figure 1**). In the following experiments, 1 × 10^6^ CFU of PA was used. At 24 or 36 h post-infection, when mice were treated with ASCs, the BALF total white blood cell count was significantly reduced (**Figure 1A**). The treatment also reduced the expression levels of TNF-α and MIP-2, and bacterial burdens in lung homogenate and BALF (**Figures 1B, C**). In addition, the pathological damage of lung tissue is significantly alleviated, accompany with lower pathological score (**Figure 1D**). In addition, no significant difference was found between 24 and 36 h.

### Stimulation of NLRC4 Inflammasome Expression Induced by PA

**Figure 2:**
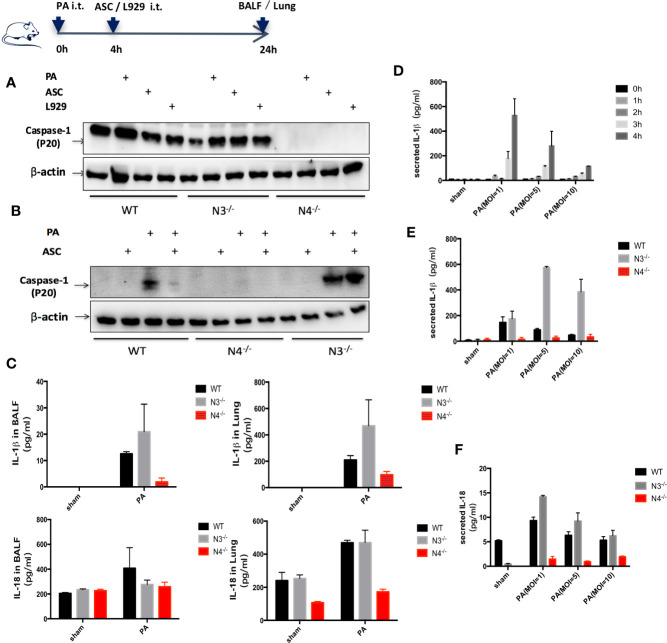
Stimulation of NLRC4 inflammasome expression induced by PA. **(A, C)** *in vivo*, compared with the control group, the expression of Caspase-1 (p20) protein, IL-18 and IL-1β were significantly increased in the PA-infected group of WT mice; Caspase-1 was scarcely expressed in each group of N4^−/−^ mice, and the expression of IL-18 and IL-1β was slightly higher in the PA-infected group than in the control group; Conversely, Caspase-1 and IL-18, IL-1β expression were significantly increased in PA-infected group of N3^−/−^ mice than in control group. In addition, the expression of Caspase-1 (p20) protein, IL-18 and IL-1β in PA+N4^−/−^ mice was significantly lower than that in PA+WT group; Compared with PA+WT group, the expression of IL-1β in PA+N3^−/−^ group was significantly increased, the expression of IL-18 and Caspase-1 (p20) protein were not significantly different. **(D)** *In vitro*, IL-1β expression level showed the highest when PA MOI was 1:1 and time was 4 h. **(B, E, F)** *In vitro*, results showed the same trend as *in vivo* experiments. Data were shown as mean ± SD. ASC, adipose tissue-derived mesenchymal stem cells; N4^−/−^, NLRC4^−/−^; N3^−/−^, NLRP3^−/−^.

A significantly increased expression of Caspase-1 (p20) protein, IL-18, and IL-1β were found in WT group infected by PA. However, only IL-1β expression was significantly increased in NLRP3^−/−^ mice. In addition, the expression of Caspase-1 (p20) protein, IL-18 and IL-1β in NLRC4^−/−^ mice was significantly lower than that in WT group (**Figures 2A, C**).

*In vitro*, peritoneal macrophages of WT mice, NLRC4^−/−^ mice and NLRP3^−/−^ mice were extracted and IL-1β expression in macrophage supernatant was detected after PA stimulation. We found that IL-1β expression level showed the highest when PA MOI was 1:1 and time was 4 h. Therefore, in the following *in vitro* study, we used MOI= 1:1 and 4 h as the parameters for PA stimulation (**Figure 2D**). *In vitro* results showed the same trend as *in vivo* experiments (**Figures 2B, E, F**).

### Inhibition of NLRC4 Inflammasome Activation by ASCs in PA-Induced Acute Pulmonary Infection

**Figure 3:**
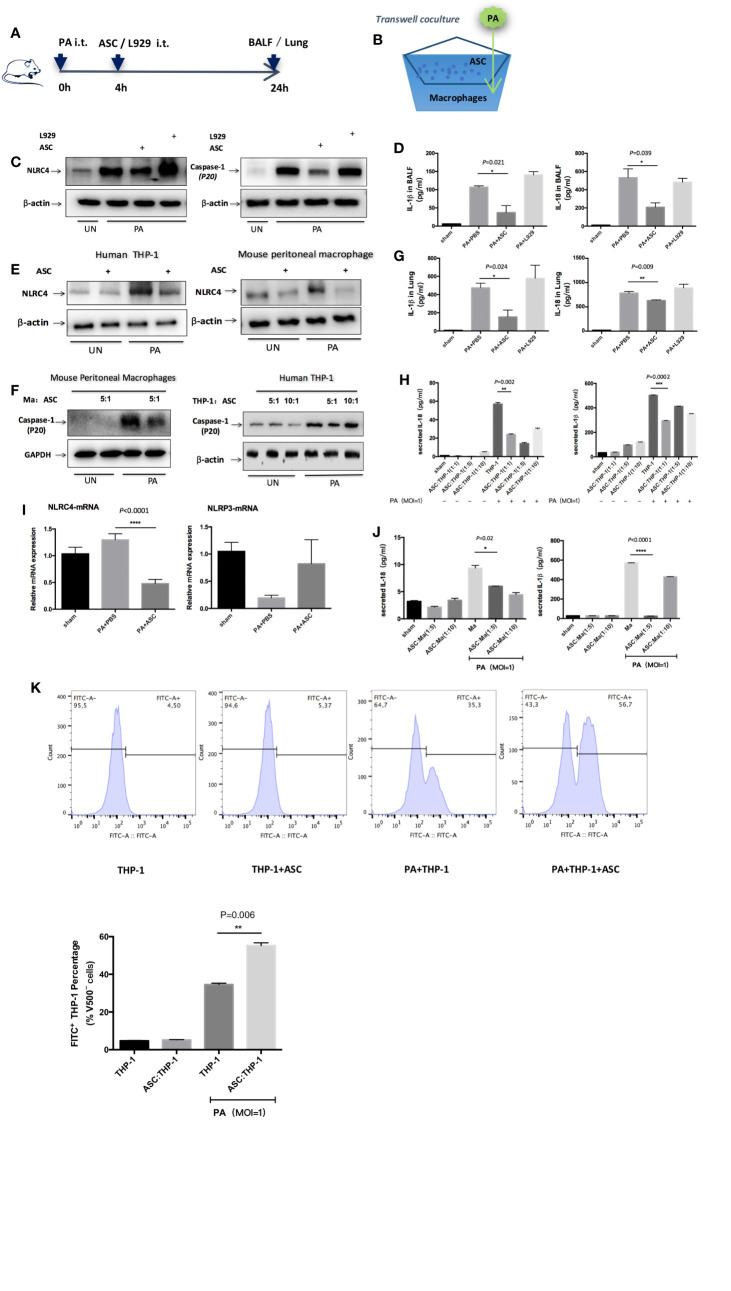
Inhibition of NLRC4 inflammasome activation by ASCs in PA-induced acute pulmonary infection. **(A)** *In vivo*, mice were infected by PA and treated with ASC or L929 cells. **(B)** *In vitro*, human THP-1 cells or mouse peritoneal macrophages were stimulated with PA for 4 h. The ASC were added to macrophages in transwell coculture system. **(C, E, F)** NLRC4 and Caspase-1 activation were both markedly suppressed in macrophages by treated or transwell coculture with ASC as assessed by Western blot. **(D, G, H, J)** Consistent with the reduction in NLRC4 and Caspase-1 activity, the levels of IL-1β and IL-18 in BALF, lung or the supernatants were all significantly reduced in ASC treatment group than in PA infection group. **(I)** PA infection induced an increase in NLRC4 mRNA in lung as measured by real time RT-PCR. Treated with ASC could affect the transcript levels of NLRC4 in lung, while NLRC4 transcripts were significantly reduced by ASC. However, NLRP3 transcripts has not been promoted by PA. Data were shown as mean ± SD. **(K)** THP-1 cells were subjected to PA stimulation and ASC co-culture *in vitro*, and the phagocytic rate of macrophages in each group was measured by flow cytometry analysis to observe the phagocytic ability of macrophages. Grouping: THP-1 untreated group (THP-1 group), ASC simple co-culture group, PA simple stimulation group and PA stimulation + ASC co-culture group. The results showed that in THP-1 cells, the phagocytic rate of macrophages in PA-simple stimulation group was increased compared to THP-1 untreated group and ASC simple co-culture group, but the phagocytic rate of PA stimulation + ASC co-culture group was significantly higher than that of PA-simple stimulation group. The phagocytosis rate in ASC simple co-culture group was not significantly different from that in THP-1 untreated group. Ma, macrophages; ASC, adipose tissue-derived mesenchymal stem cells.

The results revealed that NLRC4 and Caspase-1 activation were both markedly suppressed in macrophages by treated or transwell co-culture with ASCs as assessed by Western blot (**Figures 3A–C, E, F**). Consistent with the reduction in NLRC4 and Caspase-1 activity, the levels of IL-1β and IL-18 in BALF, lung or the supernatants were all significantly reduced in ASCs treatment group than in PA group (**Figures 3D, G, H, J**). Also, PA infection induced an increase in NLRC4 mRNA in lung as measured by real time RT-PCR. Treated with ASCs affected the transcript levels of NLRC4 in lung, while NLRC4 transcripts were significantly reduced by ASCs (**Figure 3I**). However, NLRP3 transcripts were not promoted by PA.

In addition, we observed that ASCs significantly attenuated cell pyroptosis caused by PA-stimulated activation of inflammasome (**Supplementary Figure 2**), while ASCs significantly improved the phagocytic capacity of macrophages (**Figure 3K**). To rule out the possibility that IL-1β or IL-18 might be secreted from ASCs, we stimulated cultures of ASCs alone with PA but found no detectable amount of IL-1β or IL-18 in the supernatants indicating that the NLRC4 inflammasome was not mainly activated in ASCs (**Supplementary Figure 4**).

**Figure 4:**
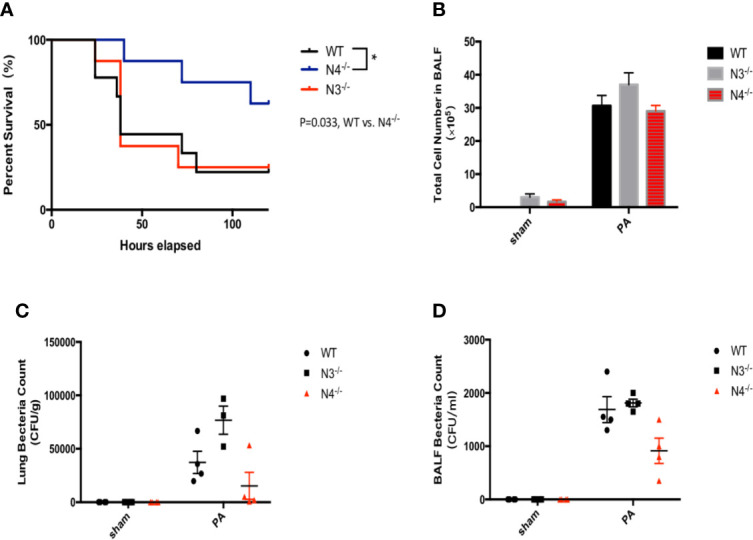
Activation of NLRC4 inflamasome induced by PA aggravated inflammatory damage in lung. **(A)** The survival rate of PA+NLRC4^−/−^ group was significantly improved compared with PA+WT group and PA+NLRP3^−/−^ group, n = 30 for all groups. **(B–D)** BALF total inflammatory cell count and bacterial load in BALF and lung in PA+NLRC4^−/−^ group were all markedly decreased. N4^−/−^, NLRC4^−/−^; N3^−/−^, NLRP3^−/−^.

To further verify that NLRC4 inflammasome may aggravate inflammatory damage in PA infection, WT mice, NLRC4^−/−^ mice, and NLRP3^−/−^ mice were infected by PA respectively. Each group was continuously observed to 120 h to record the survival of mice. The results showed that the survival rate of PA+NLRC4^−/−^ group was significantly improved compared with PA+WT group and PA+NLRP3^−/−^ group (**Figure 4A**). Also, BALF total inflammatory cell count and bacterial load in BALF and lung in PA+NLRC4^−/−^ group were all markedly decreased (**Figures 4B–D**), suggesting that activation of NLRC4 inflammasome induced by PA infection aggravated inflammatory damage in lung.

### Effect of STC-1 on NLRC4 Inflammasome Activation Induced by PA

**Figure 5:**
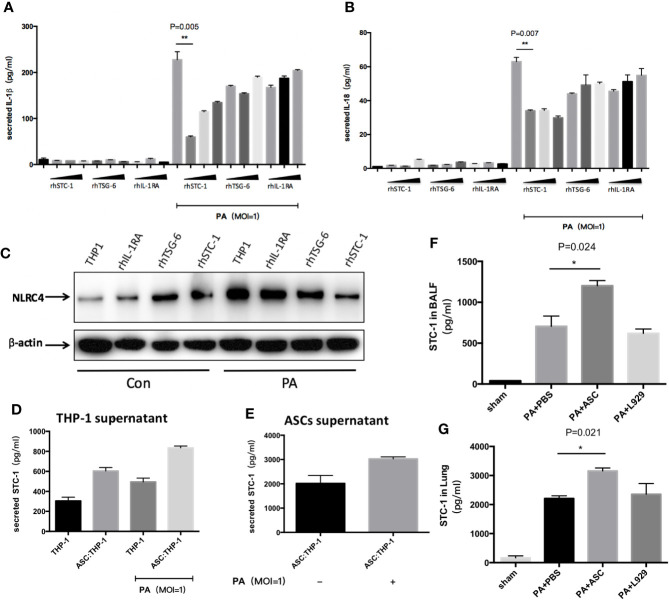
Effect of STC-1 on NLRC4 inflammasome activation induced by PA. **(A–C)** Recombinant human (rh) STC-1, (rh) TSG-6 and (rh) IL-1RA were directly and respectively added to THP-1 at PA priming step. The dose from left to right was in turn 10ng/ml、100ng/ml、200ng/ml in each recombinant group. The results demonstrated that (rh) STC-1, (rh) TSG-6 and (rh) IL-1RA dose independently decreased NLRC4 inflammasome activation as well as IL-1β and IL-18 secretion, while the inhibitory effect of (rh) STC-1was the most obvious. **(D, E)** The expression levels of STC-1 in supernatant of ASC and THP-1 were respectively examined. We found that the expression level of STC-1 in ASC supernatant in Ad-MSC co-culture group was 2017 pg/ml, and the expression of STC-1 in PA-stimulated + ASC co-culture group was significantly increased compared to ASC co-culture group (3000pg/ml). However, in THP-1 supernatant, the expression level of STC-1 in THP-1 untreated group was only 304 pg/ml. The expression level of STC-1 in PA +ASC co-culture group was significantly higher than PA stimulation group (800 pg/ml). **(F, G)** *in vivo* experiments, the expression level of STC-1 in PA+ASC treatment group was significantly higher than that in PA+PBS group. Data were shown as mean ± SD. ASC, adipose tissue-derived mesenchymal stem cells.

We detected three factor(s) that were secreted from ASCs and mediated the regulated action of ASCs on macrophages: STC-1, TSG-6 and IL-1RA. We added recombinant human (rh) STC-1, (rh) TSG-6 and (rh) IL-1RA, respectively to THP-1 at PA priming step. The results demonstrated that (rh) STC-1, (rh) TSG-6 and (rh) IL-1RA dose independently decreased NLRC4 inflammasome activation as well as IL-1β and IL-18 secretion, while the inhibitory effect of (rh) STC-1 was the most obvious (**Figures 5A–C**).

To rule out the possibility that STC-1 might be secreted from macrophages, we respectively examined the expression levels of STC-1 in the supernatant of ASCs and THP-1. We found that the expression level of STC-1 in ASCs supernatant in Ad-MSC co-culture group was 2017 pg/ml, and the expression of STC-1 in PA-stimulated + ASCs co-culture group was significantly increased compared to ASCs co-culture group (**Figure 5E**). However, in THP-1 supernatant, the expression level of STC-1 in THP-1 untreated group was only 304 pg/ml. The expression level of STC-1 in PA+ASCs co-culture group was significantly higher than PA stimulation group (**Figure 5D**), indicating that STC-1 was mainly secreted from ASCs but not macrophages in this environment which PA stimulates ASCs and THP-1 co-culture.

In addition, we observed *in vivo* experiments that the expression level of STC-1 in PA+ASCs treatment group was significantly higher than that in PA+PBS group (**Figures 5F–G**), suggesting from the side that STC-1 may be associated with the process by which ASCs attenuate PA-induced acute lung infection in mice.

## Discussion

In the present study, we evaluated the mechanisms underlying the therapeutic effect of ASCs against acute lung infection by PA. The main findings of this study can be summarized as follows: 1) PA infection attenuated macrophage phagocytosis through activation of NLRC4 inflammasome in macrophages, which eventually led to pulmonary inflammatory damage in mouse; 2) The beneficial effect of ASCs on PA-induced acute lung infection may result from their ability to suppress NLRC4 inflammasome activation, which consequently resulted in the inhibition of Caspase-1 cleavage and mature IL-1β, IL-18 secretion; 3) The increase of STC-1 production through ASCs could contribute to the inhibitory effect of ASCs on NLRC4 inflammasome activation by PA.

In this study, we have screened different doses and time points of standard PA strain (PAO1) for the optimal amount and period when setting up the rodent model of PA-induced acute lung infection. We also examined the effects of MSCs on inflammatory pathological damage in PA-infected mice. Since it is difficult to prepare sufficient numbers of BMSCs in the clinical setting, alternative sources of MSCs which are more easily harvested and expanded in a clinical setting remain needed (Fraser et al., 2006; Stolzing et al., 2008; Ikegame et al., 2011; Vidal et al., 2012; Dmitrieva et al., 2012). Currently, MSCs are isolated from adipose tissue and the umbilical cord, representing major alternative sources to bone marrow (Hoogduijn et al., 2014). In our study, we have chosen ASCs and the findings showed that ASCs were effective in reducing BALF total inflammatory cell count, bacterial loads and inflammatory markers TNF-α and MIP-2 in both lung and BALF, suggesting that ASCs efficiently reduced pathological damage caused by PA. What needs to be specifically mentioned here is the research significance of PA+PBS group design: Acute lung infection was induced by the instillation of PA intratracheally (i.t.) in our research. Three hours after instillation of PA, ASCs (resuspended with PBS) were delivered to the lungs with the same method. For the authenticity and reliability of the experiment, we additionally made PA+PBS as a control group for PA+ASCs, which means that 3 h after instillation of PA, only PBS were delivered to the lungs with the same method. The results in **Figure 1** showed that the pathogen count, cytokine and tissue injury in PA+PBS group was more significant than that in PA group, which may be due to the secondary injury to the trachea, similar to aspiration pneumonia. However, the injury in PA+ASCs group was significantly reduced, which can further indicate that ASCs have a significant effect on reducing inflammation and injury.

We, for the first time, provided the new evidence revealing that inhibition of NLRC4 inflammasome activation by ASCs significantly alleviated acute lung injury induced by PA. NLRP3 and NLRC4 inflammasomes were classified based on different structures and recognition signals and four types of inflammasomes were mostly studied among them. NLRP3 inflammasome contains pyrin domain and can be activated by LPS combined with ATP or certain bacterial toxins such as nicotin and endotoxin. NLRC4 inflammasome is a multiprotein complex comprised of the proteins NLRC4, ASCs (apoptosis-associated speck-like protein containing a caspase recruitment domain), and proCaspase-1. NLRC4 inflammasome can be activated by cytosolic flagellin of bacteria, which could be responsible for IL-1β and IL-18 processing by macrophages in response to PA infection (Sutterwala et al., 2007; Franchi et al., 2007; Miao et al., 2008). Inflammasome activation leads to the cleavage of proCaspase-1 to activated Caspase-1 that induces the processing of pro-IL-1β and pro-IL-18, which are released as mature IL-1β and IL-18. In our study, we used an intact germ PA to induce lung injury in mice. In order to evaluate whether ASCs had certain regulation on NLRC4 inflammasome in PA induced acute lung infection, we firstly performed the PA induced NLRC4^−/−^ or NLRP3^−/−^ mice and we could see in **Figure 2B** that Caspase-1 is absent in NLRC4^−/−^ but present in WT and NLRP3^−/−^ that were infected with PA. As shown in **Figure 2B**, PA mainly promoted the expression of Caspase-1 through NLRC4 pathway. So among NLRC4^−/−^ group, the expression of Caspase-1 was absent with either PA infection or ASC intervention.

Secondly, the role of activation of NLRC4 inflammasome in acute lung infections caused by PA remains controversial. Recent studies have reported that the role of NLRC4 inflammasome may perform to be complex and dualistic, both aggravating inflammation and inflammatory protection, depending on the specific pathological microenvironment in which it is located (Amer et al., 2006; Ceballos-Olvera et al., 2011; Cai et al., 2012; Faure et al., 2014). In this study, we found that the survival rate of PA+NLRC4^−/−^ mice was significantly improved compared with PA+WT mice and PA+NLRP3^−/−^ mice, suggesting that the activation of NLRC4 inflammasome may aggravate infection and increase mortality. Finally, we investigated the effects of ASCs on NLRC4 inflammasome activation against PA infection. We found that NLRC4 and Caspase-1 activation, the levels of IL-1β and IL-18 were both markedly suppressed in macrophages by ASCs. Treated with ASCs could affect the transcript levels of NLRC4 in lung. NLRC4 inflammasome activation also leads to an inflammatory cell death known as pyroptosis. We have noticed that the level of NLRP3 mRNA expression was also decreased when treated with PA infection. One possible explanation could be the expression of NLRP3 may affected by PA infection in some undefined pathways, the other reason may due to the limited sample size. In addition, we observed the effects of ASCs on the secretion of LDH in macrophage supernatants and phagocytic ability of macrophage after PA stimulation. ASCs significantly attenuated cell pyroptosis caused by PA-stimulated activation of inflammasome, while ASCs significantly improved the phagocytic capacity of macrophages. Interestingly, We have also noticed that, as shown in **Figure 2B**, although NLRP3 was knocked out in NLRP3^−/−^ group, Caspase-1 level was not completely suppressed as in WT group in addition of ASC. It has been demonstrated that severe infection injury can promote the activation of NLRC4 as well as NLRP3 inflammasome, regulate the activation of Caspase-1, and eventually lead to inflammatory injury. However, under the stimulation of different pathogens, the activation of inflammasome is different. Therefore, we assumed that MSCs may inhibit the activation of NLRC4 as well as NLRP3 to a certain extent. In NLRP3^−/−^ group, even though the NLRC4 activation pathway was mainly inhibited, the inhibition of NLRP3 downstream pathway was eliminated to a certain extent, which resulted to the expression of Caspase-1.

Multiple studies have consistently shown that the protective effects may attribute to paracrine factors secreted by MSCs. In the pathological process of acute lung injury, the immune regulation of MSCs is a key link to play an anti-inflammatory protective role, and the paracrine effect of MSCs is one of the most important mechanisms for its immune regulation (Chen et al., 2008). In our study, the effects of NLRP3 inflammasome activation related paracrine factors, such as STC-1, TSG-6, and IL-1RA (Oh et al., 2014; Fontaine et al., 2016; He et al., 2016), were discussed. The results showed that the paracrine factors STC-1, TSG-6, and IL-RA were all correlated in the inhibition of NLRC4 inflammasome by ASCs, the inhibitory effect of STC-1 on NLRC4 inflammasome seemed to be the most obvious one. The results of *in vivo* experiments also preliminarily confirmed that STC-1 may be associated with the process by which ASCs attenuate PA-induced acute lung infection in mice. However, the intrinsic molecular mechanisms of how ASCs paracrine STC-1 and act on NLRC4 inflammasome remain to be further explored.

Our study has several limitations. First, although we found ASCs negatively regulated NLRC4 inflammasome activation in part through the secretion of STC-1, the mechanisms of STC-1 on NLRC4 inflammasome remains unclear. Second, other mechanisms involving in the inhibition of NLRC4 inflammasome signaling pathways of ASCs against PA pulmonary infection remains to be investigated.

**Figure 6:**
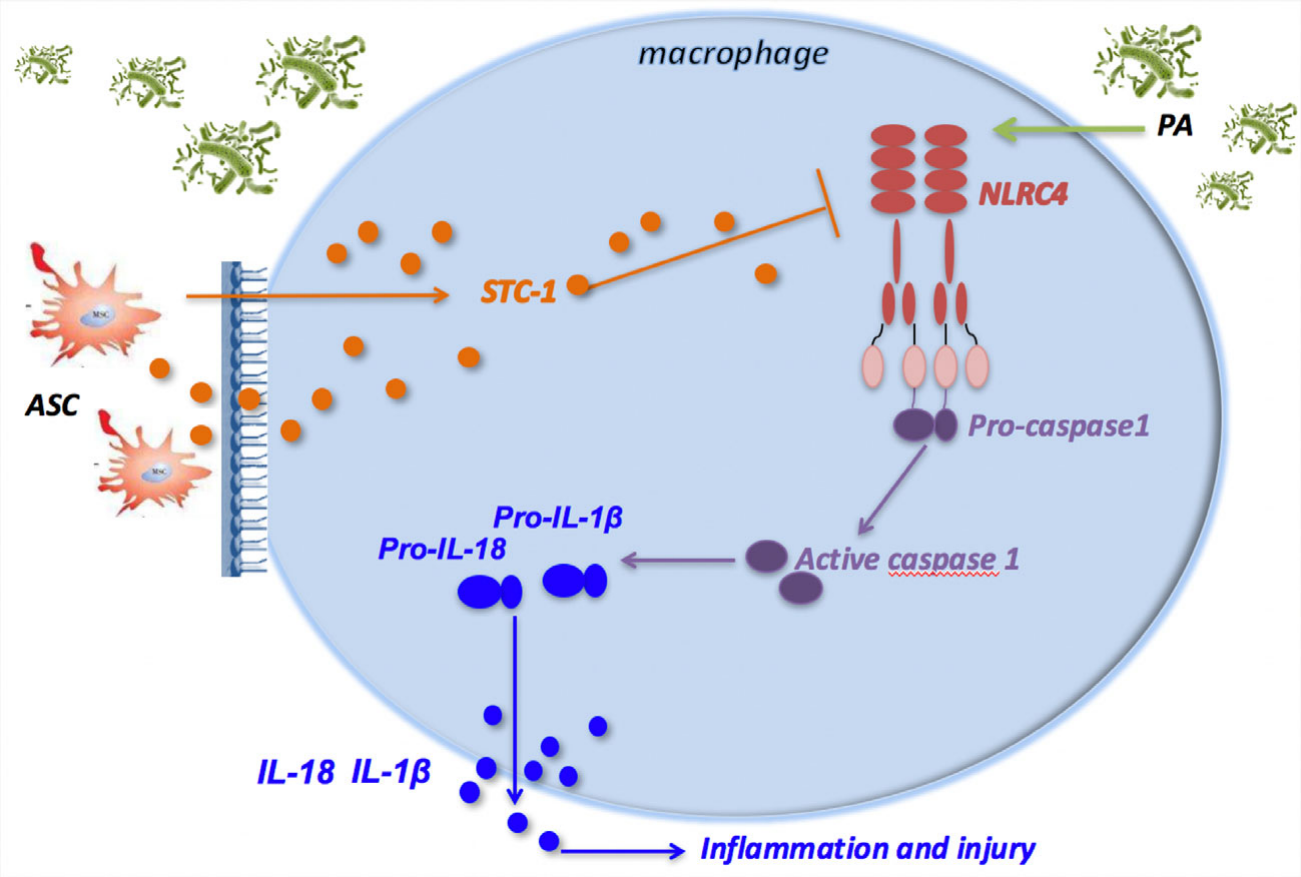
ASCs inhibit the activation of NLRC4 inflammasome in macrophages induced by PA and reduce inflammatory damage. PA infection can activate NLRC4 inflammasome in macrophage. NLRC4 Inflammasome activation leads to the cleavage of proCaspase-1 to activated Caspase-1 that induces the processing of pro-IL-1β and pro-IL-18, which are released as mature IL-1β and IL-18, leading to inflammatory damage in mouse lung tissue; ASC can negatively regulate the NLRC4 inflammasome in macrophages primarily by secreting STC‐1 in response to crosstalk with activated macrophages through PA infection, which can alleviate inflammatory damage in lung tissue of mice. ASC, adipose tissue-derived mesenchymal stem cells.

In conclusion, PA infection attenuated macrophage phagocytosis through activation of NLRC4 inflammasome in macrophages, which eventually led to pulmonary inflammatory damage in mice. ASCs reduced the activation of NLRC4 inflammasome in macrophages induced by PA infection, thereby increasing the phagocytic ability of macrophages, and ultimately improving lung tissue damage. Additionally, ASCs may inhibit NLRC4 inflammasome through the secretion of STC-1 (**Figure 6**).

## Data Availability Statement

The original contributions presented in the study are included in the article/**Supplementary Materials**. Further inquiries can be directed to the corresponding authors.

## Ethics Statement

The animal study was reviewed and approved by Animal Experimental Medicine Research Center, Ruijin Hospital, Shanghai Jiaotong University School of Medicine.

## Author Contributions

L-LL: conception and design, collection and/or assembly of data, data analysis and interpretation, manuscript writing, and provision of study material. Y-GZ and DL: conception and design, data analysis and interpretation, and manuscript writing. X-MJ: conception and design, manuscript writing, and final approval of manuscript. J-MQ: conception and design, manuscript writing, financial support, and final approval of manuscript. All authors contributed to the article and approved the submitted version.

## Funding

The study was supported by grants from the National Natural Science Foundation of China (No. 81570029 and No. 81630001), National Key Research & Development Program of China (2018YFE0102400), Shanghai Shenkang Hospital Development Center Clinical Science and Technology Innovation Project (SHDC12018102), Excellent youth talent project of Shanghai Municipal commission of health and family planning (No. 2017YQ081), Shanghai Key Discipline for Respiratory Diseases (No. 2017ZZ02014), and National Innovative Research Team of High-level Local Universities in Shanghai.

## Conflict of Interest

The authors declare that the research was conducted in the absence of any commercial or financial relationships that could be construed as a potential conflict of interest.
