# Supplementary figures and images for: Adipose-Derived Mesenchymal Stem Cells Ameliorating Pseudomonas aeruginosa–induced Acute Lung Infection via Inhibition of NLRC4 Inflammasome
Source: Front Cell Infect Microbiol. 2021 Jan 8;10:581535. doi: 10.3389/fcimb.2020.581535 (PMC7820751; doi:10.3389/fcimb.2020.581535)

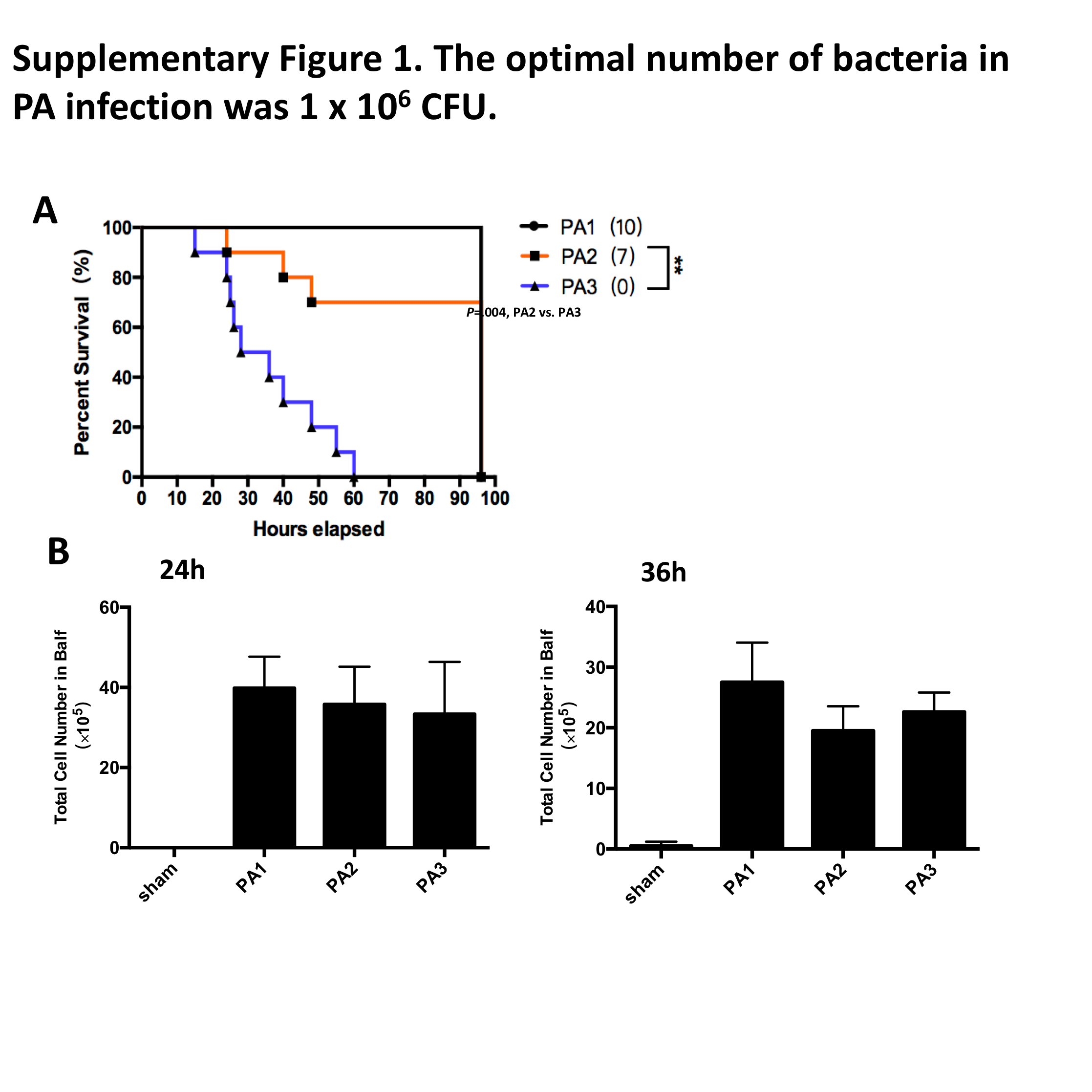

Supplement: Supplementary file 1 [file Image_1.tiff]

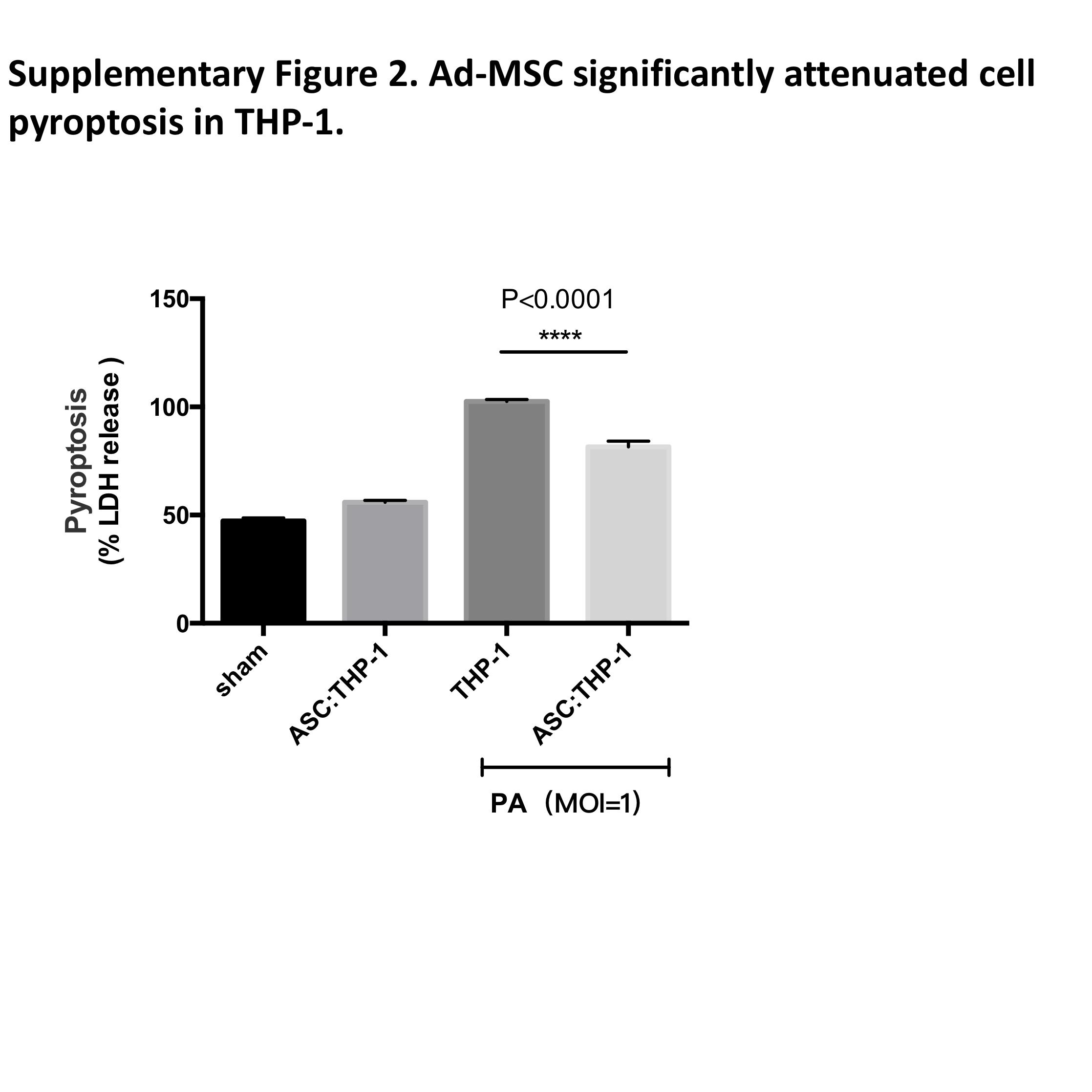

Supplement: Supplementary file 2 [file Image_2.tiff]

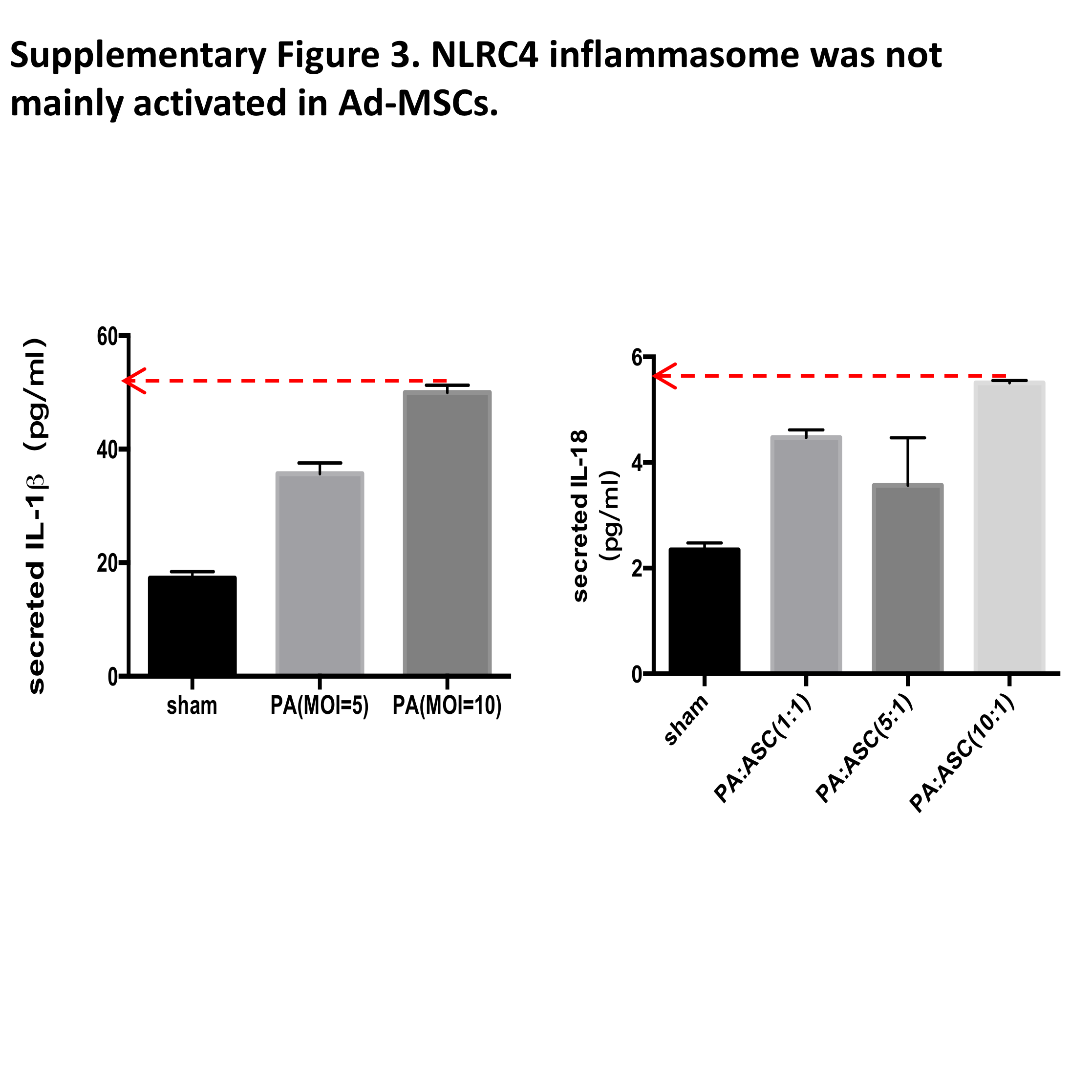

Supplement: Supplementary file 3 [file Image_3.tiff]
