# Supplementary material for: Adipose-Derived Mesenchymal Stem Cells Ameliorating Pseudomonas aeruginosa–induced Acute Lung Infection via Inhibition of NLRC4 Inflammasome
Source: Front Cell Infect Microbiol. 2021 Jan 8;10:581535. doi: 10.3389/fcimb.2020.581535 (PMC7820751; doi:10.3389/fcimb.2020.581535)
Supplement: Supplementary file 4 [file DataSheet_1.pdf]

Supplementary tables for extended numerical values and statistic results in Figures

Fig. 1A

	**24 hours (N)**	**36 hours (N)**
PA	30.05 ± 2.728 (4)	35.74 ± 3.774 (5)
PA+PBS	37.33 ± 2.19 (4)	35.38 ± 1.14 (5)
PA+ASCs	12.14 ± 2.58 (5)	5.80 ± 1.47 (5)

p= 0.7258 for PA vs. PA+PBS in 24 hours

p= 0.0002 for PA+PBS vs. PA+ASCs in 24 hours

p= 0.0201 for PA vs. PA+PBS in 36 hours

p < 0.0001 for PA+PBS vs. PA+ASCs in 36 hours

N in brackets represents as sample size for each group. Same as below.

Fig. 1B, **MIP-2 in lung:**

	**24 hours (N)**	**36 hours (N)**
PA	130.8 ± 7.920 (4)	91.45 ± 16.68 (5)
PA+PBS	117.90 ± 3.40 (4)	104.30 ± 11.88 (5)
PA+ASCs	78.91 ± 5.06 (4)	30.93 ± 4.55 (5)

p= 0.1981for PA vs. PA+PBS in 24 hours

p= 0.0007 for PA+PBS vs. PA+ASCs in 24 hours

p= 0.5938 for PA vs. PA+PBS in 36 hours

p= 0.0002 for PA+PBS vs. PA+ASCs in 36 hours

Fig. 1B, **MIP-2 in BALF:**

	**24 hours (N)**	**36 hours (N)**
PA	180.8 ± 32.17 (4)	175.4 ± 21.32 (4)
PA+PBS	217.90 ± 3.40 (4)	332.70 ± 58.33 (4)
PA+ASCs	93.91 ± 8.63 (4)	150.50 ± 5.32 (4)

p= 0.039 for PA vs. PA+PBS in 24 hours

p <0.0001 for PA+PBS vs. PA+ASCs in 24 hours

p= 0.1936 for PA vs. PA+PBS in 36 hours

p= 0.0358 for PA+PBS vs. PA+ASCs in 36 hours

Fig. 1B, TNF-α in lung:

	**24 hours (N)**	**36 hours (N)**
PA	40.77 ± 7.205 (4)	26.59 ± 7.527 (5)
PA+PBS	36.49 ± 0.64 (4)	29.23 ± 3.03 (5)
PA+ASCs	23.96 ± 2.86 (5)	3.94 ± 1.13 (5)

p= 0.006 for PA vs. PA+PBS in 24 hours

p= 0.0036 for PA+PBS vs. PA+ASCs in 24 hours

p= 0.2689 for PA vs. PA+PBS in 36 hours

p= 0.0008 for PA+PBS vs. PA+ASCs in 36 hours

Fig. 1B, TNF-α in BALF:

	**24 hours (N)**	**36 hours (N)**
PA	165.4 ± 30.65 (4)	177.9 ± 30.18 (4)
PA+PBS	242.90 ± 29.53 (4)	227.90 ± 48.22 (4)
PA+ASCs	109.30 ± 35.24 (4)	100.70 ± 3.42 (4)

p= 0.9527 for PA vs. PA+PBS in 24 hours

p= 0.0272 for PA+PBS vs. PA+ASCs in 24 hours

p= 0.4620 for PA vs. PA+PBS in 36 hours

p= 0.0390 for PA+PBS vs. PA+ASCs in 36 hours

Fig. 1C

	**in BALF (N)**	**in lung (N)**
PA	314.0 ± 49.46 (3)	10240 ± 3416 (3)
PA+PBS	332.60 ± 91.98 (5)	10847 ± 2434 (5)
PA+ASCs	12.33 ± 1.45 (3)	4348 ± 1248 (5)

p= 0.0015 for PA vs. PA+PBS in BALF in 36 hours

p=0.0401 for PA+PBS vs. PA+ASCs in BALF in 36 hours;

p= 0.7904 for PA vs. PA+PBS in lung in 36 hours

p=0.0448 for PA+PBS vs. PA+ASCs in lung in 36 hours

Fig. 1D

	**24 hours (N)**	**36 hours (N)**
PA	0.6400 ± 0.040 (3)	0.7350 ± 0.0350 (3)
PA+PBS	0.840 ± 0.040 (3)	1.010 ± 0.010 (3)
PA+ASCs	0.575 ± 0.025 (3)	0.640 ± 0.040 (3)

p= 0.63 for PA vs. PA+PBS in 24 hours

p= 0.0303 for PA+PBS vs. PA+ASCs in 24 hours

p= 0.754 for PA vs. PA+PBS in 24 hours

p= 0.0122 for PA+PBS vs. PA+ASCs in 36 hours

Fig. 2C: IL-1β:

	**in BALF (N)**	**in lung (N)**
WT	12.54 ± 1.00 (3)	209.67 ± 23.86 (3)
NLRC4$^{-/-}$	1.84 ± 1.00 (3)	97.69 ± 23.86 (3)

p= 0.0004 for WT vs. NLRC4$^{-/-}$ in BALF

p= 0.009 for WT vs. NLRC4$^{-/-}$ in lung

Fig. 2C: IL-18:

	**in BALF (N)**	**in lung (N)**
WT	407.754 ± 97.848 (3)	469.99 ± 13.81 (3)
NLRC4$^{-/-}$	259.185 ± 97.848 (3)	173.09 ± 13.81 (3)

p= 0.203536 for WT vs. NLRC4$^{-/-}$

p= 0.0002 for WT vs. NLRC4$^{-/-}$

Fig. 2E:

	PA(MOI=1) (N)	PA(MOI=5) (N)	PA(MOI=10) (N)
WT	157.88 ± 28.95 (3)	89.08 ± 12.00 (3)	48.65 ± 8.14 (3)
NLRC4$^{-/-}$	16.54 ± 28.95 (3)	19.97 ± 12.00 (3)	18.63 ± 8.14 (3)

p = 0.0081 for WT vs. NLRC4$^{-/-}$

p = 0.0045 for WT vs. NLRC4$^{-/-}$

p = 0.021 for WT vs. NLRC4$^{-/-}$

Fig. 2F:

	PA(MOI=1) (N)	PA(MOI=5) (N)	PA(MOI=10) (N)
WT	9.329 ± 0.640 (3)	6.329 ± 0.510 (3)	5.329 ± 0.510 (3)
NLRC4$^{-/-}$	1.450 ± 0.640 (3)	0.950 ± 0.510 (3)	1.950 ± 0.510 (3)

p = 0.0065 for WT vs. NLRC4$^{-/-}$

p = 0.0088 for WT vs. NLRC4$^{-/-}$

p = 0.022 for WT vs. NLRC4$^{-/-}$

Fig. 3D:

	IL-18 (N)	IL-1β (N)
PA+PBS	532.70 ± 96.16 (3)	107.30 ± 3.18 (3)
PA+ASCs	208.40 ± 47.02 (3)	37.61 ± 18.82 (3)

p = 0.0388 for PA+PBS vs. PA+ASCs in IL-18

p = 0.0217 for PA+PBS vs. PA+ASCs in IL-1β

Fig.3G:

	IL-18 (N)	IL-1β (N)
PA+PBS	785.50 ± 30.91 (3)	475.20 ± 50.61 (3)
PA+ASCs	632.00 ± 12.17 (3)	155.00 ± 75.18 (3)

p = 0.0099 for PA+PBS vs. PA+ASCs in IL-18

p = 0.0242 for PA+PBS vs. PA+ASCs in IL-1β

Fig.3H:

	**IL-18 (N)**	**IL-1β (N)**
PA+THP-1	57.00 ± 1.50 (3)	503.00 ± 2.50 (3)
PA+ASCs: THP-1(1:1)	24.06 ± 0.50 (3)	295.00 ± 1.50 (3)

p = 0.0023 for PA+ THP-1 vs. PA+ ASCs: THP-1(1:1) in IL-18

p = 0.0002 for PA+ THP-1 vs. PA+ ASCs: THP-1(1:1) in IL-1β

Fig.3J:

	**IL-18 (N)**	**IL-1β (N)**
PA+ Ma	9.33 ± 0.50 (4)	571.90 ± 0.50 (4)
PA+ ASCs: Ma(1:5)	6.02 ± 0.02 (3)	25.19 ± 0.50 (3)

p = 0.0221 for PA+ Ma vs. PA+ PA+ ASCs: Ma(1:5)

p < 0.0001 for PA+ Ma vs. PA+ PA+ ASCs: Ma(1:5)

Fig. 3I:

PA+PBS	1.299 ± 0.112 (8)
PA+ASCs	0.479 ± 0.077 (9)

p < 0.0001 for PA+PBS vs. PA+ASCs

Fig. 3K:

PA+THP-1	34.55 ± 0.7500 (3)
PA+ASCs: THP-1	55.25 ± 1.450 (3)

p =0.0062 for PA+ THP-1 vs. PA+ ASCs: THP-1

Fig. 5A-B:

	**IL-18 (N)**	**IL-1β (N)**
PA+THP-1	227.40 ± 17.56 (3)	63.00 ± 2.50 (3)
PA+ THP-1+ rhSTC-1	59.77 ± 2.25 (3)	34.06 ± 0.50 (3)

p = 0.0077 for PA+ THP-1 vs. PA+ THP-1+ rhSTC-1 in IL-18

p = 0.0052 for PA+ THP-1 vs. PA+ THP-1+ rhSTC-1 in IL-1β

Fig. 5D:

PA+THP-1	495 ± 38 (4)
PA+ASCs: THP-1	835 ± 19 (4)

p = 0.0002 for PA vs. PA+ ASCs: THP-1

Fig. 5E:

ASCs: THP-1	2017 ± 328 (5)
PA+ASCs: THP-1	3032 ± 79 (7)

p = 0.0055 for ASCs: THP-1 vs. PA+ ASCs: THP-1

Fig. 5F-G:

	**in BALF (N)**	**in lung (N)**
PA+PBS	706 ± 125 (3)	2207 ± 95 (3)
PA+ASCs	1202 ± 64 (3)	3158 ± 104 (3)

p = 0.0212 for PA+PBS vs. PA+ASCs in lung

p = 0.0243 for PA+PBS vs. PA+ASCs in BALF
